# Aromatherapy for Managing Pain in Primary Dysmenorrhea: A Systematic Review of Randomized Placebo-Controlled Trials

**DOI:** 10.3390/jcm7110434

**Published:** 2018-11-10

**Authors:** Myeong Soo Lee, Hye Won Lee, Mohamed Khalil, Hyun Suk Lim, Hyun-Ja Lim

**Affiliations:** 1 Clinical Medicine Division, Korea Institute of Oriental Medicine, Daejeon 34054, Korea; 2 Herbal Medicine Research Division, Korea Institute of Oriental Medicine, Daejeon 34054, Korea; anywon1975@gmail.com; 3 Public Health & Research Development, National Center for Complementary and Alternative Medicine, Ministry of Health, Riyadh 11662, Saudi Arabia; statkhl@hotmail.com; 4 Department of Nursing, Howon University, Gusan 54058, Korea; progress0509@hanmail.net; 5 Department of Nursing, Chodang University, Muan-gun 58530, Korea; hjlim@cdu.ac.kr

**Keywords:** aromatherapy, essential oil, inhalation, pain, dysmenorrhea, systematic review, meta-analysis

## Abstract

Aromatherapy, the therapeutic use of essential oils, is often used to reduce pain in primary dysmenorrhea. Eleven databases, including four English (PubMed, AMED, EMBASE, and the Cochrane Library) and seven Korean medical databases, were searched from inception through August 2018 without restrictions on publication language. Randomized controlled trials (RCTs) testing aromatherapy for pain reduction in primary dysmenorrhea were considered. Data extraction and risk-of-bias assessments were performed by two independent reviewers. All of the trials reported superior effects of aromatherapy for pain reduction compared to placebo (*n* = 1787, standard mean difference (SMD): −0.91, 95% CI: −1.17 to −0.64, *p* < 0.00001) with high heterogeneity (*I*^2^ = 88%). A sub-analysis for inhalational aromatherapy for the alleviation of pain also showed superior effects compared to placebo (*n* = 704, SMD: −1.02, 95% CI: −1.59 to −0.44, *p* = 0.0001, *I*^2^ = 95%). With regard to aromatherapy massage, the pooled results of 11 studies showed favorable effects of aromatherapy massage on pain reduction compared to placebo aromatherapy massage (*n* = 793, SMD: −0.87, 95% CI: −1.14 to −0.60, *p* < 0.00001, *I*^2^ = 70%). Oral aromatherapy had superior effects compared to placebo (*n* = 290, SMD: −0.61, 95% CI: −0.91 to −0.30, *p* < 0.0001, *I*^2^ = 0%). In conclusion, our systemic review provides a moderate level of evidence on the superiority of aromatherapy (inhalational, massage, or oral use) for pain reduction over placebo in primary dysmenorrhea.

## 1. Introduction

Dysmenorrhea is characterized by lower abdominal pain that occurs during menstruation [1,2]. The prevalence rate in women is not precise, although systematic reviews estimate a prevalence ranging from 16 to 95% [2].

Several pharmacological interventions are available for the management of dysmenorrhea [1,2]. Current evidence shows that non-steroid anti-inflammatory drugs (NSAIDs) are beneficial for reducing pain in primary dysmenorrhea compared to placebo controls [2,3]. However, there are safety issues associated with using NSAIDs including gastrointestinal discomfort, hemorrhage, and cardiovascular risks [2,3]. Simple analgesics (aspirin, paracetamol) are likely beneficial for alleviating pain in the short term but may also have potential adverse events (AEs) including skin reactions [2,3]. Combined oral contraceptives may also provide effective management for dysmenorrhea, but they also exhibit AEs including irregular uterine bleeding and the induction of endometriosis [2,4]. Many women try CAM as an alternative or complementary therapy to conventional drug therapy for healthcare [5]. One of the main reasons is to avoid the AEs of conventional treatments [5,6].

Aromatherapy, the use of essential oils for a therapeutic purpose, is a popular type of CAM in the UK [5]. Essential oils can be absorbed via olfaction, through the external skin, internal skin, and ingestion, and the applications are divided into inhalation, topical use, and oral use [7]. Several pediatric academic medical centers in USA utilize aromatherapy for pain, or anxiety surrounding procedures [8,9]. Some textbooks have also reported the beneficial effects of aromatherapy for a wide range of conditions including infection, insomnia, pain, inflammation, stress, mental health, cancer, and women’s health [10,11]. However, some of the proposed therapeutic effects of aromatherapy are not well-supported by clinical studies [7]. An overview suggested that aromatherapy may induce relaxation and improve pain and psychological health [12]. Two recent systematic reviews of aromatherapy massage for pain in dysmenorrhea was published during the performance of our systematic review, and they showed superior effects of aromatherapy massage on alleviating pain in primary dysmenorrhea compared to placebo oil massage [13,14]. However, these reviews have several limitations including the lack of a comprehensive search and the lack of inclusion of recent trials despite a publication date within the current review. Furthermore, one review included only an aromatherapy massage, whereas aromatherapy can be used as an inhalation therapy or orally in addition to massage [13]. The other review failed to pool all available data and the effects may be exaggerated with bias [14]. Currently, no comprehensive systematic reviews have investigated the efficacy of various types of aromatherapy for pain management in primary dysmenorrhea.

The aim of this study was to summarize and critically assess the available evidence regarding the efficacy of aromatherapy in managing pain in primary dysmenorrhea.

## 2. Methods

### 2.1. Data Sources

The following databases were searched from their inception through August 2018: PubMed, AMED, EMBASE, the Cochrane Library, and seven Korean medical databases (Korean Studies Information, DBPIA, Oriental Medicine Advanced Searching Integrated System, Research Information Service System, KoreaMed, the Town Society of Science Technology, and the Korean National Assembly Library). The search terms used were as follows: (aromatherapy OR essential oil OR aroma inhalation OR lavender OR rose) AND (dysmenorrhea OR menstruation disturbances OR menstruation disorders OR menstrual disorder OR pelvic pain OR painful menstruation OR painful period OR period pain OR primary dysmenorrhea) AND (randomized controlled trial) in Korean and English. We used the search terms for specific oils after pilot screening the databases (lavender and rose). In addition, the reference lists of all the retrieved articles were hand-searched for further relevant literature. Hard copies of all the included articles were read in full. Because all of the various databases used for this study possessed their own subject headings, each database was searched independently.

### 2.2. Study Selection

#### 2.2.1. Type of Studies

All randomized controlled trials (RCTs) and quasi-RCTs were included. Observational, cohort, case-control, case series, and qualitative studies, uncontrolled trials, and laboratory studies were excluded.

#### 2.2.2. Type of Participants

Women of reproductive age with primary dysmenorrhea, i.e., individuals with no identifiable pelvic pathology as indicated by a pelvic examination, ultrasound scans, and laparoscopy, and women self-reporting a diagnosis of primary dysmenorrhea were included. The exclusion criteria consisted of identifiable pelvic pathology and dysmenorrhea resulting from the use of an intra-uterine contraceptive device.

#### 2.2.3. Types of Interventions

We included those trials that used aroma inhalation, aromatherapy massage, and oral use of aromatherapy with any type of essential oil. Aromatherapy is defined as the therapeutic use of essential oils from plants. There was no limitation on the number of essential oils used, the dosage, the forms of aromatherapy, or the duration of treatment.

#### 2.2.4. Types of Comparisons

We compared placebo controls to aromatherapy used alone or in combination with massage.

### 2.3. Outcome Measures

The primary outcome was pain, specifically a reduction in pain (i.e., menstrual pain) that occurred only during the intervention or as a result of the intervention measured by a visual analogue scale (VAS), by other validated scales, or as a dichotomous outcome. The secondary outcome was AEs.

### 2.4. Data Extraction and Risk-of-Bias Assessment

All articles were read by two independent reviewers (HWL & HJL) who extracted the data according to the pre-defined criteria. Information regarding the participants, interventions, outcomes, and results were obtained from each report. Any disagreement between the two authors was resolved by discussion. Another author (MSL) acted as an arbiter for unresolved disagreements. The Iranian papers published in Persian were sent to a professional company for translation into English. The risk-of-bias (ROB) was assessed using the following six criteria from the Cochrane risk of bias tool [15]: (1) random sequence generation, (2) allocation concealment, (3) blinding of participants and personnel, (4) blinding of outcome assessment, (5) incomplete outcome data, and (6) selective outcome reporting. This review used “L,” “U,” and “H” as judgement keys: “Low” indicated a low risk of bias (L), “Unclear” indicated that the risk of bias was uncertain (U), and “High” indicated a high risk of bias (H). Disagreements were resolved by discussion amongst all reviewers.

### 2.5. Data Synthesis

All statistical analyses were conducted using the Cochrane Collaboration’s software program, Review Manager (RevMan), Version 5.3.0 for Windows (The Nordic Cochrane Center, Copenhagen, Denmark). Differences between the intervention and control groups were assessed. We used the generic inverse variance method to analyze the standardized mean difference (SMD) with 95% confidence intervals (CIs) between the two groups. We used the generic inverse variance method in RevMan to analyze the difference in means between the two groups. For studies with insufficient information, we contacted the primary authors to acquire and verify data when possible. If appropriate, we then pooled the data across the studies using random effect models. The chi-square test for heterogeneity and the *I*^2^ test were used to evaluate the heterogeneity of the included studies. We aimed to perform subgroup analyses according to the type of aromatherapy. We also investigated reporting bias using a funnel plot.

### 2.6. Assessing the Overall Quality of the Evidence

We evaluated the overall quality of the evidence for pain using the Grading of Recommendations Assessment, Development, and Evaluation GRADEproGDT (online version, available at https://gradepro.org/). The GRADE approach considers five reasons, namely, risk of bias, imprecision, inconsistency, indirectness, and publication bias, for downgrading the overall quality of evidence. We presented the main findings of the review in a tabular format.

## 3. Results

### 3.1. Description of the Included Trials

**Figure 1:**
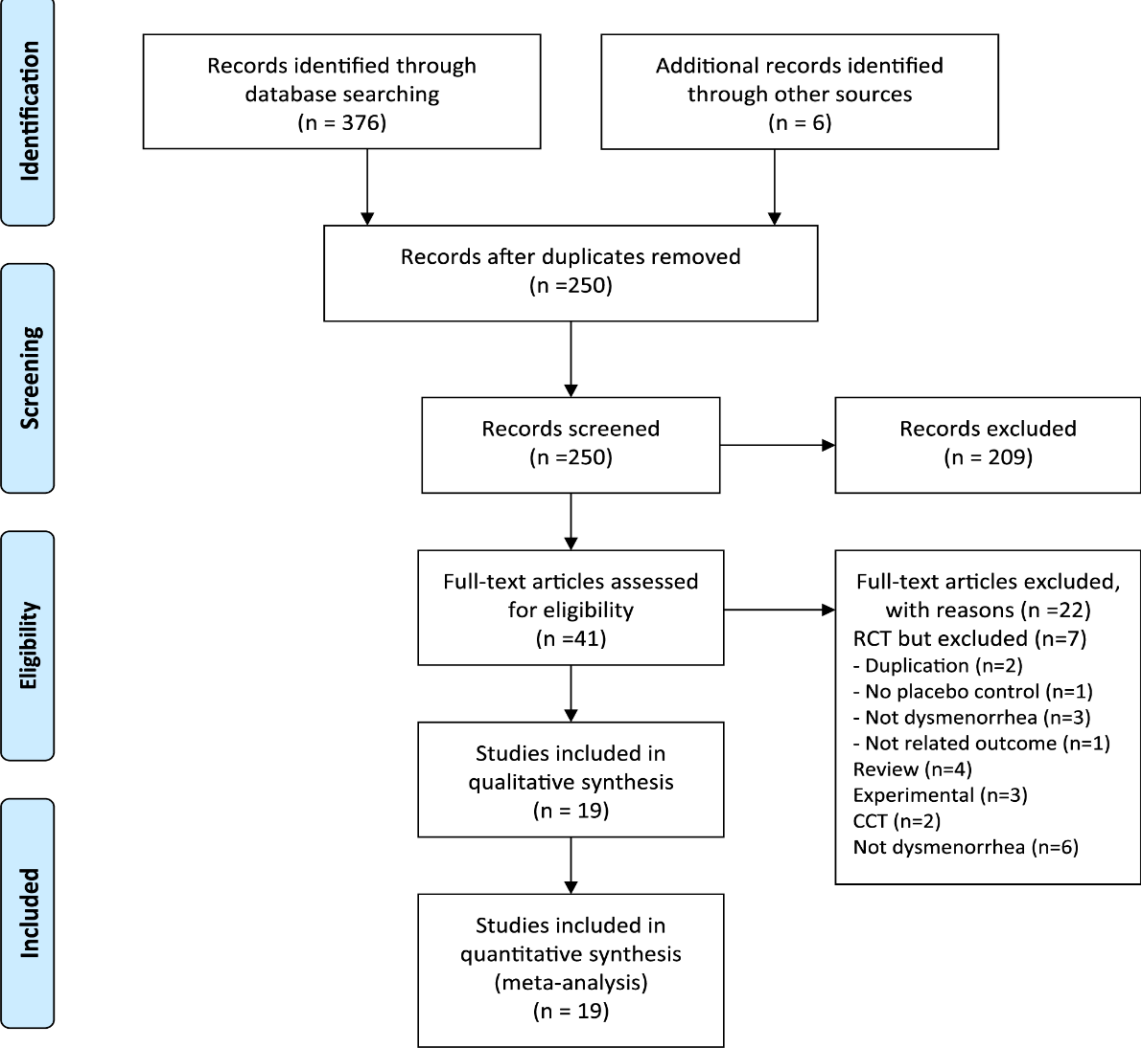
Flow diagram of selection process. CCT: non-randomized controlled trial; RCT: randomized controlled trial.

We identified 382 potentially relevant studies, 19 of which met our inclusion criteria (Figure 1). The key data are summarized in Table 1 [16,17,18,19,20,21,22,23,24,25,26,27,28,29,30,31,32,33,34]. Seven RCTs tested inhalation aromatherapy [16,17,18,19,20,21,22], 11 RCTs assessed aromatherapy with massage [19,23,24,25,26,27,28,29,30,31,32] (one RCT compared massage and inhalation [19]), and the other two studies investigated oral aromatherapy [33,34]. Eleven trials originated from Iran [17,18,20,21,22,23,25,26,29,33,34], 3 from Korea [19,30,31], 2 from Turkey [16,24], 2 from Egypt [27,28], and 1 from Taiwan [32]. Fifteen of the included trials used a parallel design [16,17,18,19,20,21,22,25,26,27,29,30,31,32,34], whereas four RCTs employed a crossover design [23,24,28,33]. Aromatherapy was used once in 4 trials [16,26,30,33] and more than twice in the remaining trials [17,18,19,20,21,22,23,24,25,27,28,29,31,32,34]. Seven RCTs were registered in the trial registration in Iran [17,18,22,25,26,29,34]. Three of these studies were registered during recruiting [18,22,26], whereas four were registered retrospectively [17,25,29,34].

### 3.2. Risk of Bias

**Figure 2:**
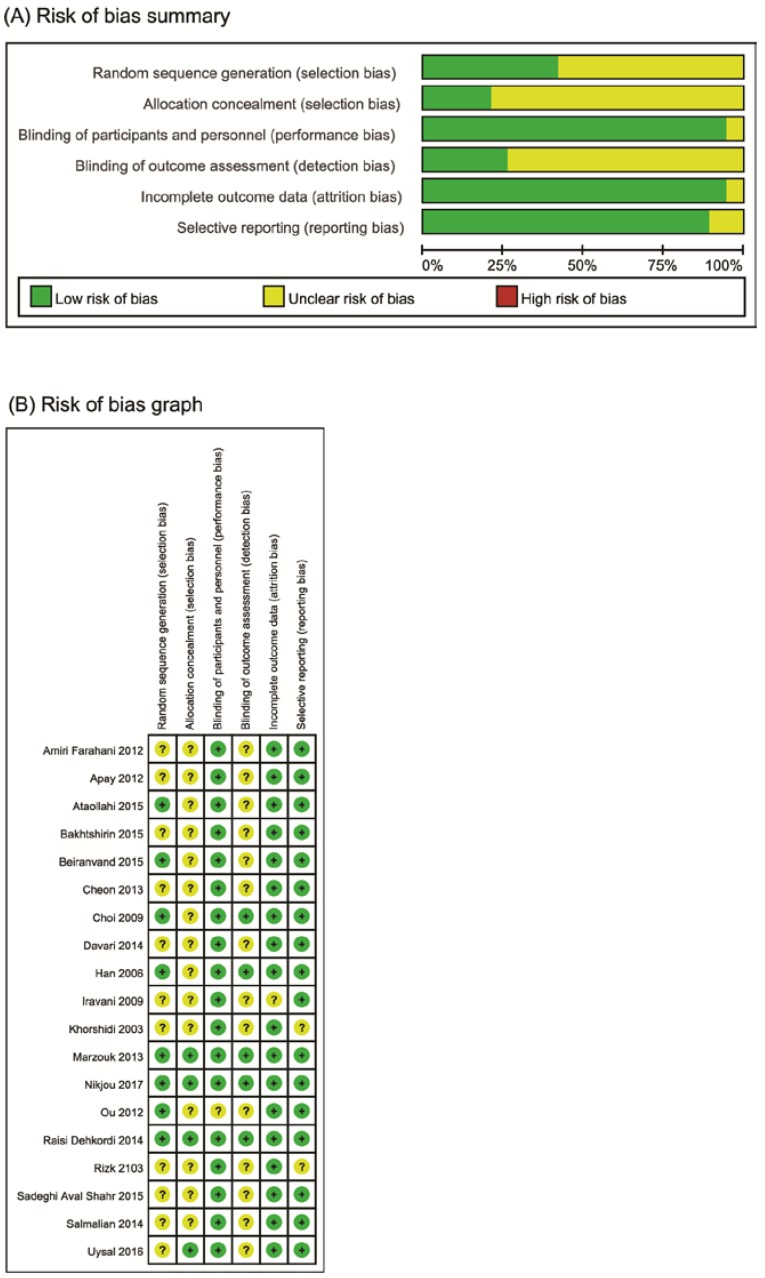
(**A**) Risk of bias graph: review authors’ judgements about each risk of bias item presented as percentages across all included studies. (**B**) Risk of bias summary: review authors’ judgements about each risk of bias item for each included study. +: low risk of bias; −: high risk of bias; ?: unclear risk of bias.

The risk of bias was moderate across several domains in the included trials (Figure 2). Eight trials used adequate sequence generation [17,18,19,25,28,30,32,34], and only four described allocation concealments [16,17,18,28]. All trials employed potential blinding methods, whereas only five used blinding of the outcome assessment [17,18,19,28,30]. All included studies reported complete outcome data and selective outcomes.

### 3.3. Outcome Measurements

#### 3.3.1. Pain

**Figure 3:**
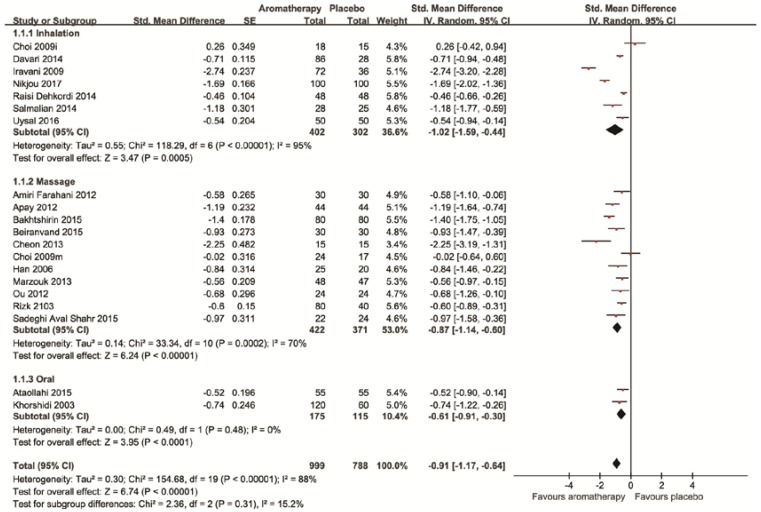
Forest plot of effects of aromatherapies on pain of primary dysmenorrhea.

All of the 19 RCTs tested the effects of aromatherapy for treating dysmenorrhea compared to the placebo. All the trials reported superior effects of aromatherapy for pain reduction compared to the placebo. The meta-analysis also showed the superior effects of aromatherapy on pain reduction (*n =* 1787, SMD: −0.91, 95% CI: −1.17 to −0.64, *p* < 0.00001) with high heterogeneity (*I*^2^ = 88%, Figure 3) A sub-analysis for inhalational aromatherapy for the alleviation of pain also showed superior effects compared to the placebo (*n =* 704, SMD: −1.02, 95% CI: −1.59 to −0.44, *p* = 0.0001, *I*^2^ = 95%, Figure 3) [16,17,18,19,20,21,22]. Regarding aromatherapy massage, the pooled results of 11 studies showed favorable effects of aromatherapy massage on pain reduction compared to placebo aromatherapy massage (*n =* 793, SMD: −0.87, 95% CI: −1.14 to −0.60, *p* < 0.00001, *I*^2^ = 70%, Figure 3) [19,23,24,25,26,27,28,29,30,31,32]. Oral aromatherapy had superior effects compared to the placebo (*n =* 290, SMD: −0.61, 95% CI: −0.91 to −0.30, *p* < 0.0001, *I*^2^ = 0%, Figure 3) [33,34].

#### 3.3.2. Adverse Events

Only two trials assessed AEs [26,30], whereas the other 17 did not. No adverse reactions were reported in these two studies [26,30].

#### 3.3.3. Reporting Bias

**Figure 4:**
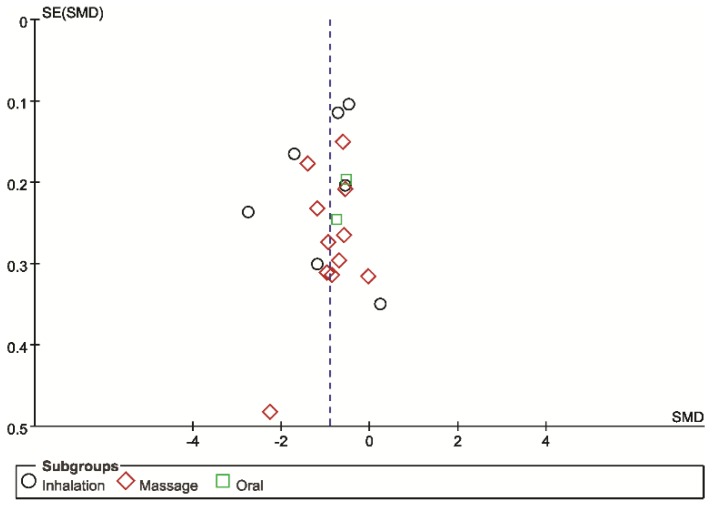
Funnel plot for aromatherapies for pain management compared with placebo aromatherapies.

Funnel plots were asymmetrical for the SMD of pain, showing potential publication bias (Figure 4).

## 4. Discussion

Several rigorous RCTs have investigated the non-specific effects of aromatherapy on reducing the symptoms of primary dysmenorrhea. Our systematic review and meta-analysis provides suggestive evidence of the superiority of three types of aromatherapy including inhalation, massage, and oral use for the treatment of pain associated with dysmenorrhea over placebo controls. The level of evidence for this finding was moderate (Table 2); therefore, aromatherapy can be recommended for use in clinical practice for managing dysmenorrhea.

Compared to a recently published systematic review of aromatherapy massage only [13], we identified a total of 13 new RCTs [16,17,18,19,20,21,22,25,27,29,31,33,34] (4 evaluated massages [25,27,29,31]) that assessed all types of aromatherapy and have comprehensively updated the evidence for aromatherapy. For aromatherapy massage, the results are the same as those of a previous systematic review showing that aromatherapy massage may be effective for pain reduction in primary dysmenorrhea [13]. We also successfully included 10 more RCTs [18,20,21,24,25,27,29,33,34] compared with another review [14]. Our results showed a smaller effect size of aromatherapy compared to their results. A pooling of small numbers of trials may exaggerate the treatment effects. The newly added trials offer supportive evidence for aromatherapy treatment for lower abdominal pain in primary dysmenorrhea compared to the placebo control, regardless of the type of aromatherapy. 

One trial that used a three-armed parallel design failed to show superior effects of aromatherapy (inhalation and massage) for reducing pain based on VAS scores in primary dysmenorrhea [19]. However, dysmenorrhea symptoms, which were the primary outcome of this study, were significantly reduced by the use of aromatherapy inhalation (*n =* 30, SMD=−2.56 (−3.52, −1.61), *p* < 0.0001), whereas they were not affected by aromatherapy massage (*n =* 41, SMD = −0.29 (−0.91, 0.34), *p* = 0.36) [19]. Therefore, all of the included studies support the effects of aromatherapy for the management of primary dysmenorrhea.

Our review shows that inhalation is the most effective type of aromatherapy because “inhaling essential oils is the fastest method of getting the essential oils into the body.” There are two ongoing or unpublished trials that were registered in the Iranian Registry of Clinical Trials (IRCT) (http://www.irct.ir/): One used aromatherapy inhalation (rosa damascena and bitter orange, *n =* 90) (IRCT2016031113940N3), and the other employed aromatherapy massage (geranium essence, *n =* 90) (IRCT2017013132329N1) to test the effects of aromatherapy for dysmenorrhea compared to the placebo. These results did not seem to change the direction of the effects, but they did increase the level of evidence.

The duration of the interventions varied across the trials. Four trials used only a single treatment, and the results showed that aromatherapy has use for the acute management of pain in primary dysmenorrhea [16,26,30,33]. Four trials used a crossover design to test the effects of aromatherapy [23,24,28,33]. Three employed a one-cycle wash-out period [23,24,28], whereas the cycles were unclear for one study [33]. Residual effects may not exist, but it is unclear because none of the included studies performed follow-up assessments. Future studies should provide evidence of any long-lasting effects of this intervention.

Only eight trials reported the use of random sequence generation methods [17,18,19,25,28,30,32,34], and only four of the included trials used allocation concealment [16,17,18,28]. We included only placebo-controlled trials and evaluated low ROB in the blinding of participants and personal. Although the authors claimed that their studies were single-, double-, or triple-blinded in design, due to the nature of aromatherapy, it may be difficult to deceive the participants because of the smell of essential oils. In addition, two studies employed self-aromatherapy massage, and the participants may have known their assignment group [26,32]. No studies assessed the success of blinding, and we cannot completely elucidate the failure of blinding. With regard to subjective outcomes such as pain, inadequate sequence generation, blinding, and allocation concealment are likely to produce exaggerated effects of the interventions [35]. The high heterogeneity of the meta-analysis may also be due to these limitations [35]. However, the included trials generally had poor quality reporting, and we could not obtain sufficient information regarding the exact methods used from their papers. Therefore, the real ROB may be different from the ROB that was evaluated by the published papers. Future studies should follow the recommended reporting guidelines for trials such as CONSORT and CONSORT extensions.

Although the pooled effect sizes ranged from moderate to high, there are several issues regarding the use of aromatherapy for managing the pain of primary dysmenorrhea in real clinical practice. One of these issues is the lack of a standardized treatment procedure in each study. Aromatherapy is delivered via several methods including massage, inhalation, and oral use and is also often composed of a blend of several essential oils from many herbs. Therefore, there is extensive heterogeneity in the ingredients, dose, and delivery methods between the studies included in this review. In this situation, the standardization of materials and doses used in clinical trials is one of the most necessary factors for demonstrating good reproducibility of research results for real clinical practice.

Cochrane systematic review suggested a low level of evidence that using non-steroidal anti-inflammatory drug (NSAID) is effective for managing dysmenorrhea [3]. However, there are no clinical trials comparing any type aromatherapy with essential oils now. Some trials have used aromatherapy as an adjunct to NSAID, but not in head-to-head comparison. It may be hard to perform comparative studies in the CAM field and in countries with dual medical systems including Korea and Taiwan.

The mechanism of action of essential oils might pose formidable challenges. One plausible explanation might be the involvement of the analgesic component of essential oils including linalool for lavender, menthol for peppermint, and fenchone for fennel. [10]. Another explanation might involve the parasympathetic nervous system related to touch and smell [10]. Further basic research is needed to fully understand the mechanism of aromatherapy with essential oils.

AEs are another important issue for using essential oils with humans. However, only two studies have assessed AEs, and none are reported in this review. One systematic review reported that “aromatherapy may lead to AEs which can occasionally be serious” [36], and another recent article suggested that potential risks may exist because aromatherapy essential oils are a source of aggregate exposure to skin allergens [37]. Although not relevant to this population, recent studies showed the possibility that lavender causes prepubertal gynecomastia in boys [38,39]. Although the small number of included trials assessed AEs, there is still a possibility of AEs in relation to the oral ingestion of essential oils, tropical uses for possible skin reactions, and inhalation for respiratory infection or reactions. Further study is needed to clarify this.

Our study has several limitations. First, most of the included trials were performed in Middle East and East Asian countries. The generalization of these results to other countries might be limited. Secondly, we attempted to retrieve possible eligible studies through comprehensive searches of numerous databases regardless of publication language, but there may have been missed trials related to this topic. Furthermore, there are some concerns about the fact that the search terms only included certain types of essential oils: lavender and rose. We searched again after finishing the review with additional terms such as peppermint and fennels, but no additional eligible studies were found. Recently, many trials related to aromatherapy have been published in Middle Eastern countries, specifically Iran. The potential obstacles to assessing local countries can prevent the retrieval of all trials. The funnel plot shows the possible publication bias, and the lack of registration and retrospective registration threaten the validity of the included studies. Overall, potential reporting biases including location, language bias, and publication bias can distort the complete picture of the evidence.

## 5. Conclusions

This systematic review provides a moderate level of evidence for the efficacy of aromatherapy (inhalation, massage, and oral use) in alleviating pain in primary dysmenorrhea. However, the total number of RCTs included in the analysis and their ROB limit us from drawing firm conclusions. Future RCTs seem warranted; however, they must overcome the methodological drawbacks of the current studies.

## Figures and Tables

**Table 1:** Summary of randomized placebo-controlled trials of aromatherapy for dysmenorrhea (pain).

First Author (Year) [Ref] Country	Sample Size; Age (years); Severity	Intervention	Control	Treatment Duration	Result	Comments Design/AEs
Uysal (2016) [16] Turkey	105 19–30 Pain on VAS ≥5	(A) Rose oil (Inhalation, <1 m above patients, *n* = 52), plus diclofenac sodium (75 mg)	(B) Placebo (saline, <1 m above patients, *n* = 53), plus diclofenac sodium (75 mg)	1 time (10–30 min)	*p* < 0.01 in favor of aromatherapy	Diclofenac 75 mg Treatment time is not clear Parallel/n.r.
Raisi Dehkordi (2014) [17] Iran	96 18–28 Mild or moderate (AMVMS)	(A) Lavender (Inhalation, 7–10 from the nose, rub their palm with aroma oil, *n =* 48)	(B) Placebo (sesame oil, same methods with A, *n =* 48)	5 min (1 h after experiencing dysmenorrhea) every 6 h for the 1st 3 days of menstruation, one or two treatments in 2 consecutive menstrual cycle	*p* < 0.01 in favor of aromatherapy	Not validated questionnaire Retrospective registration Parallel/n.r.
Nikjou (2017) [18] Iran	200 19–30 n.r.	(A) Lavender (Inhalation, 3 drops, *n =* 100)	(B) Placebo (diluted milk, same methods with A, *n =* 100)	30 min (0.5 h after experiencing dysmenorrhea) for the 1st 3 days of menstruation, one treatment in 2 consecutive menstrual cycle	*p* < 0.01 in favor of aromatherapy	Registration while recruiting Parallel/n.r.
Choi (2009) [19] Korea	74 not clear Pain on VAS ≥7	(A) Lavender (Inhalation, *n =* 18) (B) Lavender (Massage abdomen, *n =* 24)	(C) Placebo (vitamin C, same methods with A, *n =* 15) (D) Placebo (Massage, almond oil, *n =* 17)	Wearing necklace for the daytime during the menstruation Massage was done 10 min once a day for 1 menstrual cycles	NS	Parallel/n.r.
Davari (2014) [20] Iran	150 18–29 Mild or moderate MDQ	(A) Lavender (Inhalation, *n =* 28) (B) Rosemary (Inhalation, *n =* 29) (C) Lavender and rosemary (Inhalation, *n =* 29)	(D) Placebo (*n =* 30) (E) *mefenamic acid* (*n* = 28) ^†^	Twice daily for 3 days the start of menstruation	*p* < 0.001, in favor of aromatherapy	Parallel/n.r.
Iravani (2009) [21] Iran	108 18–24 Moderate to severe	(A) Zataria Multiflora (Inhalation, 1%, 25 drops, *n =* 36) (B) Zataria Multiflora (Inhalation, 2%, 25 drops, *n =* 36)	(C) Placebo (Inhalation, 25 drops, *n =* 36)	25 drops every 4 h from the beginning of pain for 3 cycles	*p* < 0.001 in favor of aromatherapy	Parallel/n.r.
Salmalian (2014) [22] Iran	84 18–24 Mild or moderate	(A) Thymus vulgaris (Inhalation, 25 drops, *n =* 28), plus placebo capsule	(B) Placebo (Inhalation, n.r., *n =* 28), plus placebo capsule (C) Ibuprofen (200 mg capsule, *n* = 28), plus placebo oil	25 drops every 6 h on 1st day of menstrual cycle and the beginning of pain for 2 consecutive cycles	*p* < 0.001 in favor of aromatherapy	Triple-blind but no reporting of details Registration while recruiting Parallel/n.r.
Bakhtshirin (2015) [23] Iran	80 18–24 Pain on VAS ≥6	(A) Lavender oil (Massage abdomen, *n =* 80)	(B) Placebo (Massage abdomen, *n =* 80)	15 min for 1 cycle (2nd and 3rd cycle) by nursing and midwifery students	*p* < 0.001 in favor of aromatherapy	1 cycle washout period Cross-over/n.r.
Apay (2012) [24] Turkey	44 20.3 ± 1.1 Pain on VAS ≥6	(A) Lavender oil (Massage abdomen, *n =* 44)	(B) Placebo (Massage odorless liquid petrolatum, *n =* 44)	15 min for 1 cycle (2nd and 3rd cycle)	*p* < 0.001 in favor of aromatherapy	1 cycle washout period Cross-over/n.r.
Beiranvand (2015) [25] Iran	60 18–26 Pain on VAS >5	(A) Lavender oil (Massage, 2 drops in 2.5 cc of almond oil, 15 min, *n =* 30)	(B) Placebo (Massage with almond oil only, *n =* 30)	48 h before and after menstruation. Massage was done 15 min twice a day for two menstrual cycles above the pubic area	*p* < 0.001 in favor of aromatherapy	Retrospective registration Parallel/n.r.
Sadeghi (2015) [26] Iran	75 18–35 Pain on VAS ≥5	(A) Rose oil (Massage, *n =* 25)	(B) Placebo (Massage, almond oil, unscented, *n =* 25) (C) *Massage only* (*n* = 25) ^†^	1 time, 5 drops, 15 min on the 1st day of menstruation Self-massage	*p* < 0.005 in favor of aromatherapy	Registration while recruiting Parallel/None
Rizk (2013) [27] Egypt	120 17–21 Moderate to severe	(A) Peppermint oil (Massage abdomen, *n =* 40) (B) Ginger oil (massage, *n =* 40)	(C) Placebo (Massage abdomen, almond, *n =* 95)	15 min daily for 5 days for 2 cycles	*p* < 0.001 in favor of aromatherapy	Parallel/n.r.
Marzouk (2013) [28] Egypt	95 17–20 Pain on VAS ≥6	(A) Essential oils (cinnamon, clove, rose and lavender, massage, *n =* 95)	(B) Placebo (Massage abdomen, sweet almond, *n =* 95)	Once daily, 10 min for 7 days before menstruation by a researcher in a student clinic	*p* = 0.006 in favor of aromatherapy	1 cycle washout period Cross-over/n.r.
Amiri Farahani (2012) [29] Iran	90 21 verbal multi-dimensional scoring system ≥2	(A) Essential oils (Massage, lavender oil 2% (2 drops), peppermint oil 2% (2 drops) in 4 mL of almond oil, *n =* 30)	(B) Placebo (Massage, almond oil, *n =* 30) (C) *Massage only* (*n* = 30) ^†^	A week before the start of menstrual cycle until the presence of pain, massage at the top of pubic area for 15 min per day. To be repeated for two cycles	*p* = 0.03 in favor of aromatherapy	Retrospective registration Parallel/n.r.
Han (2006) [30] Korea	75 19–26 Pain on VAS ≥6	(A) Essential oils (Massage abdomen, lavender, clary sage, rose, *n =* 25)	(B) Placebo (Massage abdomen, almond oil, *n =* 25) (C) *No treatment* (*n* = 25) ^†^	1 time, 15 min	*p* = 0.008 in favor of aromatherapy	Parallel/None
Cheon (2013) [31] Korea	30 Pain on VAS ≥6	(A) Essential oils (Massage abdomen, nutmeg, fennel, marjoram, *n =* 15)	(B) Placebo (Massage abdomen, almond oil, *n =* 15)	3 mL, 10 min, once a day from 14 days before menstruation to the starting date of next menstruation	*p* < 0.001 in favor of aromatherapy	Parallel/n.r.
Ou (2012) [32] Taiwan	48 Over 18 Pain on VAS ≥5	(A) Essential oils cream (Massage abdomen, cream, lavender, clary sage, marjoram, massage, *n =* 24)	(B) Placebo (Massage abdomen, cream, synthetic fragrance, massage, *n =* 24)	From the end of the last menstruation continuing to the beginning of the next menstruation Self-massage	*p* = 0.02 in favor of aromatherapy	Compare the data of 1st cycle (pre) with the data of 2nd cycle (post) Pain measured with NRS did not show significance Parallel/n.r. Pain (VRS)
Khorshidi (2003) [33] Iran	60 17–25 Mild or moderate (AMVMS)	(A) Fennel oil (Oral, 1%, *n =* 60) (B) Fennel oil (Oral 2%, *n =* 60)	(C) Placebo (n.r., *n =* 60)	1 time, administrated as soon as pain felt	(1) *p* < 0.05 in favor of aromatherapy (A and B) (2) *p* < 0.05 in favor of aromatherapy (A and B)	n.r. in details of treatment n.r. in washout period Cross-over/n.r. Pain (LS)
Ataollahi (2015) [34] Iran	110 21.4 Moderate to severe pain (VAS ≥5)	(A) Rosaceous extract (Oral, *n =* 55)	(B) Placebo (n.r., *n =* 55)	10 drops twice daily or the first three days of the cycle for two consecutive cycles	*p* = 0.001 in favor of aromatherapy	Retrospective registration Parallel/n.r. Pain (VRS)

AMVMS: Andersch’s and Milsom’s verbal multi-dimensional scoring system; LS: Likert scale; MDQ: menstrual distress questionnaire; n.r.: not reported; NS: not significant; VAS: visual analogue scale; VRS; verbal rating scale. ^†^ These controls were not placebo group and we excluded them from analysis.

**Table 2:** Summary of findings.

Aromatherapy Compared to Placebo for Pain in Primary Dysmenorrhea				
Patient or population: patients with pain in primary dysmenorrhea Intervention: Aromatherapy Comparison: placebo Outcomes: Pain				
Intervention vs. comparator	Anticipated absolute effects * (95% CI)		No. of Participants (studies)	Quality of the evidence (GRADE)
	Risk with placebo	Risk with Aromatherapy		
Aromatherapies vs. Placebo		The SMD of in the intervention groups was 0.91 standard deviations lower (1.17 to 0.64 lower)	1787 (19 studies)	⊕⊕⊕⊝ moderate ^a,b^
Aromatherapy inhalation vs. Placebo		The SMD in the intervention groups was 1.02 standard deviations lower (1.59 to 0.44 lower)	704 (7 studies)	⊕⊕⊕⊝ moderate ^a,b^
Aromatherapy massage vs. Placebo		The SMD of pain in the intervention groups was 0.87 standard deviations lower (1.14 to 0.6 lower)	793 (11 studies)	⊕⊕⊕⊝ moderate ^a,b^
Aromatherapy oral use vs. Placebo		The SMD of pain in the intervention groups was 0.61 standard deviations lower (0.91 to 0.3 lower)	290 (2 studies)	⊕⊕⊕⊝ moderate ^a^
GRADE Working Group grades of evidence: High quality: We are very confident that the true effect lies close to that of the estimate of the effect. Moderate quality: We are moderately confident in the effect estimate: The true effect is likely to be close to the estimate of the effect, but there is a possibility that it is substantially different. Low quality: Our confidence in the effect estimate is limited: The true effect may be substantially different from the estimate of the effect. Very low quality: We have very little confidence in the effect estimate: The true effect is likely to be substantially different from the estimate of effect.				

* The risk in the intervention group (and its 95% confidence interval) is based on the assumed risk in the comparison group and the relative effect of the intervention (and its 95% CI). CI: confidence interval; SMD: standardized mean difference. ^a^ Lack of randomization sequence generation and allocation concealment; ^b^ High heterogeneity.
